# Supplementary material for: Emotional discourse analysis of COVID-19 patients and their mental health: A text mining study
Source: PLoS One. 2022 Sep 16;17(9):e0274247. doi: 10.1371/journal.pone.0274247 (PMC9481002; doi:10.1371/journal.pone.0274247)
Supplement: Attachment — Submitted filename: Response to Reviewer comments.docx [file pone.0274247.s001.docx]

**Response Letter**

Dear Reviewers and Dr. Carrà,

We appreciate the interest that you have taken in our manuscript entitled “Emotional Discourse Analysis of COVID-19 Patients and their Mental Health: An NLP based Study”. The comments are very insightful and valuable for improving our manuscript. We have made revisions in accordance with the reviewers’ enlightening suggestions. Revised and rewritten portions are marked in dark blue in the revised manuscript. We have also included the point-by-point response to the reviewer’s comments shown below (Pages 1-19 to Reviewer 1; Pages 20-26 to Reviewer 2).

Thank you for your interest and efforts and we are looking forward to hearing from you in due time regarding our manuscript, and we are ready to respond to any further questions and comments you may have.

Thanks and best wishes,

Authors

**Response to Reviewer Comments**

**Reviewer #1:**

In the manuscript entitled “Emotional Discourse Analysis of COVID-19 Patients and their Mental Health: An NLP based Study”, the authors aimed to study potential differences in the emotional status between people who reported COVID-19 infection and healthy controls, benefiting from novel approaches based on natural language processing methods.
The topic is very interesting and the specific methodological approach appears promising in the mental health field, possibly contributing to explore emotional status and the potential link with a differential mental health burden.
**Response:** we are grateful to the reviewer for the positive comments.

Nevertheless, some changes are required to improve the manuscript quality:

**• In the Introduction section, the study justification should be clearly presented, by focusing on the need to explore mental health burden or at least proxies of it (** [**https://doi.org/10.1016/S0140-6736(21)02143-7**](https://doi.org/10.1016/S0140-6736(21)02143-7) **). Similarly, in the Abstract, at least one sentence is needed to describe the study justification.**

**Response:** Thanks for this constructive comment. We have added a paragraph to justify the need to explore mental health burden at the beginning of Introduction on page 1, as copied below:

“The emergence of the COVID-19 (coronavirus disease 2019) has raised significant questions about the mental health burden of the population relating to social restrictions, lockdowns, school and business closures, loss of livelihoods, decreases in economic activity, and shifting priorities of governments in their attempt to control COVID-19. Notably, COVID-19 may result in significant physical and mental consequences among those who become infected. While the physical treatment modalities for COVID-19 have received extensive attention, the need for exploration of the mental health impact of SARS-CoV-2 infection on patients has never been more urgent [3].”

We have also added two sentences in the abstract:

“COVID-19 has caused negative emotional responses in patients, with significant mental health consequences for the infected population. The need for an in-depth analysis of the emotional state of COVID-19 patients is imperative.”

Reference

3. Santomauro, Damian F et al. Global prevalence and burden of depressive and anxiety disorders in 204 countries and territories in 2020 due to the COVID-19 pandemic. The Lancet, 398, 10312, 1700- 1712. https://doi.org/10.1016/S0140-6736(21)02143-7

**• In the Materials and Methods section, the authors should better clarify the nature of the study (e.g., cross-sectional design). When were narratives collected for people who experienced COVID-19? The authors may wish to take this into account also in the Discussion section.**

**Response:** We have added a paragraph in the Study Design section to clarify the nature of the study on page 3, as copied below:

“The present study combined qualitative and quantitative approaches in data collection and text analysis. The narrative data emerged from a larger cross-sectional project, i.e., “Lived-Experience Narratives and Mental Health of People in China during the COVID-19 Pandemic” [11-12, 22, 29]. The project used semi-structured interviews to collect narrative discourses concerning lived experiences of COVID-19 in China, from the period from 30 June 2020 to 31 January 2021. The project teams recruited participants by convenience sampling.”

References

11. Deng, Y.; Wang, L.; Yang, J.; Xie, L.; Chen, Y. How covid-19 patient narratives concerning reinfection mirror their mental health: A case series. Psychiatria Danubina 2021, 33(1), 114-119.
12. Deng, Y.; Yang, J.; Wang, L.; Chen, Y. How lived experiences of COVID-19 shape mental health: A case series of COVID-19 patients from Wuhan, China. Alpha Psychiatry 2021, 22(5),269-274.
22. Deng, Y.; Li, H.; Park, M. Emotional Experiences of COVID-19 Patients in China: A Qualitative Study. Int. J. Environ. Res. Public Health 2022, 19, 9491. <https://doi.org/10.3390/ijerph19159491>
29. Deng, Y.; Yang, J.; Wan, W. Embodied metaphor in communication about lived experiences of the COVID-19 pandemic in Wuhan, China. PLOS ONE 2021, 16(12), e0261968.

**• Further details should be added in terms of sampling strategies and potentially sample size calculations with additional comments in the Limitations section, where appropriate.**

**Response:** This study adopted convenience sampling, which was clarified on page 3, as shown below:

“The project teams recruited participants by convenience sampling. The COVID-19 patients were randomly recruited from Chongqing Public Health Medical Center, China”.

As the sample size was small, we acknowledged it in *Study limitations and future research* section on pages 16-17, which reads as:

“This study has several limitations. First, we collected the narrative discourses of 34 COVID-19 patients and 24 healthy controls by convenience sampling. Our sample size was small and not entirely balanced, and our findings may thus not be generalizable to large populations. Furthermore, the interviews were conducted during two different waves of the COVID-19 pandemic within China, namely summer and autumn-winter, across 2020-2021. The dynamic change of emotional status among the two groups across the two periods may have long-term effects.…….”

“…In light of these challenges, we hope and anticipate that similar future investigations could draw on larger sample sizes of patient narratives. Enquires along this line can control the intervals of different interviews and compare emotional symptoms of COVID-19 patients across different periods….”

**• Similarly, NLP should be described more in detail, thus providing the reader with additional information regarding the methodological approach for features computation. For example, the authors should report whether they benefited from a specific software (and related packages) and they should add a more in-depth description of chosen techniques. In addition, they should clarify whether they combined qualitative and quantitative approaches.**

**Response:** we have added a whole section “Measures” to report the methodological approach for features computation on pages 7-9, as copied below:

**Measures**

**……**

***Analysis of generic features of emotional narratives***

“In the present study, generic features of emotional narratives, including Type-Token ratio, mean word-length, average number of words per sentence, number of sentences per narrative, and number of words per narrative were measured among patients and healthy controls. The Type-Token ratio shows the number of unique words divided by the total number of words in the emotional narratives corpus. A lower ratio indicates more use of the same words in the text. Mean word-length refers to the average number of Chinese characters per word. Longer words are usually more complex and indicates better use of language. Regarding average number of words per sentence, longer sentences tend to be syntactically more complex and express more complex ideas. As for number of sentences per narrative, more sentences in a specific emotional narrative of similar length indicate the use of shorter, less complex sentences. Total number of words per narrative specifies word frequency in a specific emotional narrative [21].

The emotional narratives corpus was loaded into Python for generic feature analysis, drawing on ‘*CorpusReader* ‘class in the NLTK (Natural Language Toolkit)—an open source Python library for natural language processing [33]. This package made file opening and stream control robust, and lent support to large-scale text retrieving and operating of the current corpus. As *CorpusReader* class in NLTK provides various text processing measurements (e.g., *paras()*, *sents()*, *words()*), it was used to automatically measure grammatical categories, such as word, sentence, and paragraph, in specific narratives of patients and controls (with *fileids*, *categories* argument). In this regard, average X per Y = len(X) / len(Y) was computed with various sub-functions of *CorpusReader* classes including *reader.words()*, *reader.sents()*, *reader.paras()* in conditions such as *reader.words (categories=’patient’)*. ”

***Analysis of frequency of emotional words***

“To compute sentiment statistics, we used the package called ‘*CategorizedCorpusReader.words’ (categories=’group’)* in NLTK to calculate frequency of emotional words from specific group of interest (e.g., patient or control, positive or negative, HAPPY or SAD). First, words with no substantive meaning (functional words, punctuations) were filtered out, based on the stopwords list of Harbin Institute of Technology [32]. Next, frequency of emotional polarity and 5/21 categories of emotional words were extracted according to the Chinese Affective Lexicon ontology [30], by utilizing *‘probability.ConditionalFreqDist’* class in NLTK. By doing so, a conditional frequency distribution table was generated. *ConditionalFreqDist* (CFD) is essentially word frequency data stored in *key:value* pairs, where ‘*key’* is the user-defined condition and ‘*value’* is word frequency distribution (a *‘FreqDist’* class) of a group satisfying the condition. CFD is able to create individual ‘*FreqDist*’ objects with specific conditions of various sentiments (i.e., polarity, primary, or subcategorized emotions) and source of observation (i.e., patient or control).

Utilizing these tools, we conducted sentiment analysis by selecting the frequency of emotional vocabulary for each group according to different conditions. For instance, the occurrence of the vocabulary group of the primary emotion ‘*DISGUST’* among healthy controls was retrieved with the query (*CFD['control']['disgust']*). Other emotional types were measured in a similar way (e.g., *CFD [‘patient’][‘NC’]* calculated the frequency of fear related words among the patient group). Apart from searching for individual observation values, we used the plot function in *FreqDist* and the Python library, ‘*Matplotlob’,* to visualize the frequency of different types of emotional words by groups.”

***Analysis of significant word identity features of emotional narratives***

“Significant word identity features, such as the probabilistic tendency of specific words, can mark mental status of COVID-19 patients as compared to healthy controls. In this study, the Log-likelihood ratio (LL) was adopted to measure the difference in word identity between the two sub-corpora. Table 3 shows the rationales of the comparison and mathematical formula [34-36] that were implemented via ‘*AntConc’* software. Table 4 shows a comparison of word identity in SAD narratives between the two matched groups. Looking at ‘not’ and ‘no’ in Table 4, the high LL value suggests that patients narrated more negative experiences or attitudes compared to healthy controls. The same is true of words such as *‘isolation’, ‘days’, ‘want’, ‘worry’,* and *‘infectious’,* which concerned patients’ personal experiences of infection, quarantine, and treatment. In contrast, words such as ‘*Wuhan’*, ‘*life’*, ‘*world’*, and ‘*patient’* were used more frequently in health controls compared to the patient group, suggesting that healthy controls were more concerned with external circumstances such as the community and the country rather than themselves. ”

**Table 3.** Rationale of Log-likelihood ratio (LL) for word identity comparison [34-36]

|  | Patient  Narratives (i*) | Control  Narratives (i*) | Total |
| --- | --- | --- | --- |
| Frequency of Target Word (O*) | a | b | a+b |
| Frequency of other words | c-a | d-b | c+d-a-b |
| Total (*N) | c | d | c+d |

$$-2\ln\lambda=2\sum_{i} O_{i} \ln\left( \frac{O_{i}}{E_{i}} \right), where E_{i}=\frac{N_{i}\sum_{i} O_{i}}{\sum_{i} N_{i}}$$

**Table 4.** Most distinctive words of SAD narratives between the two matched groups

| Word Feature | Log- likelihood | Patient (N=34) | | Control (N=24) | | Over/ Underuse |
| --- | --- | --- | --- | --- | --- | --- |
|  |  | **Frequency** | **Relative Frequency** | **Frequency** | **Relative Frequency** |  |
| *then* | 52.53 | 178 | 0.63% | 226 | 1.30% | - |
| *not* | 48.14 | 609 | 2.16% | 224 | 1.29% | + |
| *Wuhan* | 46.30 | 11 | 0.04% | 48 | 0.28% | - |
| *anyway* | 43.98 | 70 | 0.25% | 4 | 0.02% | + |
| *possibly* | 42.66 | 79 | 0.28% | 123 | 0.71% | - |
| *isolation* | 41.81 | 80 | 0.28% | 7 | 0.04% | + |
| *no* | 39.47 | 160 | 0.57% | 34 | 0.20% | + |
| *for sure* | 32.94 | 61 | 0.22% | 5 | 0.03% | + |
| *days* | 29.95 | 130 | 0.46% | 29 | 0.17% | + |
| *want* | 28.81 | 192 | 0.68% | 55 | 0.32% | + |
| *life* | 27.14 | 9 | 0.03% | 32 | 0.18% | - |
| *sister* | 25.99 | 27 | 0.10% | 0 | 0.00% | + |
| *world* | 25.98 | 1 | 0.00% | 17 | 0.10% | - |
| *patient* | 25.64 | 7 | 0.02% | 28 | 0.16% | - |
| *worry* | 25.57 | 34 | 0.12% | 1 | 0.01% | + |
| *infectious* | 25.02 | 26 | 0.09% | 0 | 0.00% | + |

Note: LL values of 15.13 or higher are significant (d.f.=1, p<0.0001)

In the ***study design*** section on pages 3-4, we have added illustration about how qualitative and quantitative approaches were combined in the present study, as copied below:

Study Design

“The present study combined qualitative and quantitative approaches in data collection and text analysis. The narrative data emerged from a larger cross-sectional project, i.e., “Lived-Experience Narratives and Mental Health of People in China during the COVID-19 Pandemic” [11-12, 22, 29]. The project used semi-structured interviews to collect narrative discourses concerning lived experiences of COVID-19 in China, from the period from 30 June 2020 to 31 January 2021.”【page 3】

……

“The lived experience narrative discourse was then annotated qualitatively according to the five primary emotions [21] and the 21 emotional subcategories in the Chinese Affective Lexicon Ontology [30]. In the discourse that follows, the generic features of the emotional narratives (i.e., mean word-length, words per sentence, sentences per narrative, and words per narrative), frequency of emotional words, and significant word identity features between the two matched groups were computed quantitatively by the text-mining method.”【page 4】

References

11. Deng, Y.; Wang, L.; Yang, J.; Xie, L.; Chen, Y. How covid-19 patient narratives concerning reinfection mirror their mental health: A case series. Psychiatria Danubina 2021, 33(1), 114-119.
12. Deng, Y.; Yang, J.; Wang, L.; Chen, Y. How lived experiences of COVID-19 shape mental health: A case series of COVID-19 patients from Wuhan, China. Alpha Psychiatry 2021, 22(5),269-274.
21. Hong, K.; Nenkova, A.; March, M. E.; Parker, A. P.; Verma, R.; Kohler, C. G. Lexical use in emotional autobiographical narratives of persons with schizophrenia and healthy controls. Psychiatry Research 2015, 225(1-2),40-49.
22. Deng, Y.; Li, H.; Park, M. Emotional Experiences of COVID-19 Patients in China: A Qualitative Study. Int. J. Environ. Res. Public Health 2022, 19, 9491. <https://doi.org/10.3390/ijerph19159491>
29. Deng, Y.; Yang, J.; Wan, W. Embodied metaphor in communication about lived experiences of the COVID-19 pandemic in Wuhan, China. PLOS ONE 2021, 16(12), e0261968.
30. Xu, L.; Lin, H.; Pan, Y.; Ren, H.; Chen, J. Constructing the affective lexicon ontology. Journal of the China Society for Scientific and Technical Information 2008, 27(2), 180-185.
32. Che, W.; Feng, Y.; Qin, L.; Liu, T. N-LTP: An open-source neural language technology platform for Chinese. Available online: https://arxiv.org/abs/2009.11616 (accessed on 27 February 2022).
33. Bird, S; Klein, E; Loper, E. *Natural Language Processing with Python*. O’Reilly Media Inc, 2009. <https://www.nltk.org/book/>
34. Williams, K. The failure of Pearson's goodness of fit statistic. Journal of the Royal Statistical Society. 1976, 25(1) 49.
35. Rayson, P. E. Matrix: A statistical method and software tool for linguistic analysis through corpus comparison. Lancaster University, 2003.
36. Rayson, P., Garside R. Comparing corpora using frequency profiling. In The Workshop on Comparing Corpora. 2000, 1-6.

**• In the current version of the manuscript, the study design paragraph includes some details that are specific of the chosen methodological approach. Therefore, it would be useful to add a reference to the paragraph in which the authors describe the current approach in detail.**

**Response:** we have added the details and reference regarding the chosen methodological approach on pages 3-4, as copied below:

“Due to the COVID-19 prevention policies, telephone interviews were employed to ensure the personal safety of the interviewer and participants [20]. Participants’ narrative discourses of lived experiences involved multiple dimensions of their emotional and mental health status. The term “lived experiences” designates the phenomenological tradition regarding experiences of the everyday-life world. Such lived experiences are prereflective and less available to our awareness [14]. Thus, the lived experience narratives is capable of capturing areas of participants’ mental health status that cannot be explored by the survey method [12, 14]”

References:

13. Deng, Y.; Yang, J.; Wang, L.; Chen, Y. How lived experiences of COVID-19 shape mental health: A case series of COVID-19 patients from Wuhan, China. Alpha Psychiatry 2021, 22(5),269-274.
14. Missel, M.; Bernild, C.; Christensen, S.W.; Dagyaran, I.; Berg, S.K. It’s Not just a virus! Lived experiences of people diagnosed with COVID-19 infection in Denmark. Qualitative Health Research 2021, 31(5), 822-834.
20. Wu, C.; Cheng, J.; Zou, J.; Duan, L.; Campbell, J. E. Health-related quality of life of hospitalized COVID-19 survivors: An initial exploration in Nanning city, China. Social Science & Medicine 2021, 274, 113748.

**• More importantly, a subparagraph Measures should be added to the Materials and Methods section.**

**Response:** As the point before the above one illustrated, we have added a whole “Measures” section to report the methodological approach for features computation on pages 7-9, as copied below:

**Measures**

“Based on the preprocessed corpus labeled with emotional tags, quantitative analysis of the emotional narrative followed. The first step involved automated analysis of generic features of the emotional narratives of COVID-19 patients and healthy controls, including mean word-length, words per sentence, sentences per narrative, and words per narrative. In the second step, sentiment analysis was implemented by calculating the frequency of emotional words. In doing so, differences regarding polarity and intensity of emotions were exposed between patients and controls. Lastly, significant features of word identity across the emotional narratives were compared between the two matched groups. ”

***Analysis of generic features of emotional narratives***

“In the present study, generic features of emotional narratives, including Type-Token ratio, mean word-length, average number of words per sentence, number of sentences per narrative, and number of words per narrative were measured among patients and healthy controls. The Type-Token ratio shows the number of unique words divided by the total number of words in the emotional narratives corpus. A lower ratio indicates more use of the same words in the text. Mean word-length refers to the average number of Chinese characters per word. Longer words are usually more complex and indicates better use of language. Regarding average number of words per sentence, longer sentences tend to be syntactically more complex and express more complex ideas. As for number of sentences per narrative, more sentences in a specific emotional narrative of similar length indicate the use of shorter, less complex sentences. Total number of words per narrative specifies word frequency in a specific emotional narrative [21].”

“The emotional narratives corpus was loaded into Python for generic feature analysis, drawing on ‘*CorpusReader* ‘class in the NLTK (Natural Language Toolkit)—an open source Python library for natural language processing [33]. This package made file opening and stream control robust, and lent support to large-scale text retrieving and operating of the current corpus. As *CorpusReader* class in NLTK provides various text processing measurements (e.g., *paras()*, *sents()*, *words()*), it was used to automatically measure grammatical categories, such as word, sentence, and paragraph, in specific narratives of patients and controls (with *fileids*, *categories* argument). In this regard, average X per Y = len(X) / len(Y) was computed with various sub-functions of *CorpusReader* classes including *reader.words()*, *reader.sents()*, *reader.paras()* in conditions such as *reader.words (categories=’patient’)*. ”

***Analysis of frequency of emotional words***

“To compute sentiment statistics, we used the package called ‘*CategorizedCorpusReader.words’ (categories=’group’)* in NLTK to calculate frequency of emotional words from specific group of interest (e.g., patient or control, positive or negative, HAPPY or SAD). First, words with no substantive meaning (functional words, punctuations) were filtered out, based on the stopwords list of Harbin Institute of Technology [32]. Next, frequency of emotional polarity and 5/21 categories of emotional words were extracted according to the Chinese Affective Lexicon ontology [30], by utilizing *‘probability.ConditionalFreqDist’* class in NLTK. By doing so, a conditional frequency distribution table was generated. *ConditionalFreqDist* (CFD) is essentially word frequency data stored in *key:value* pairs, where ‘*key’* is the user-defined condition and ‘*value’* is word frequency distribution (a *‘FreqDist’* class) of a group satisfying the condition. CFD is able to create individual ‘*FreqDist*’ objects with specific conditions of various sentiments (i.e., polarity, primary, or subcategorized emotions) and source of observation (i.e., patient or control).

Utilizing these tools, we conducted sentiment analysis by selecting the frequency of emotional vocabulary for each group according to different conditions. For instance, the occurrence of the vocabulary group of the primary emotion ‘*DISGUST’* among healthy controls was retrieved with the query (*CFD['control']['disgust']*). Other emotional types were measured in a similar way (e.g., *CFD [‘patient’][‘NC’]* calculated the frequency of fear related words among the patient group). Apart from searching for individual observation values, we used the plot function in *FreqDist* and the Python library, ‘*Matplotlob’,* to visualize the frequency of different types of emotional words by groups.”

***Analysis of significant word identity features of emotional narratives***

“Significant word identity features, such as the probabilistic tendency of specific words, can mark mental status of COVID-19 patients as compared to healthy controls. In this study, the Log-likelihood ratio (LL) was adopted to measure the difference in word identity between the two sub-corpora. Table 3 shows the rationales of the comparison and mathematical formula [34-36] that were implemented via ‘*AntConc’* software. Table 4 shows a comparison of word identity in SAD narratives between the two matched groups. Looking at ‘not’ and ‘no’ in Table 4, the high LL value suggests that patients narrated more negative experiences or attitudes compared to healthy controls. The same is true of words such as *‘isolation’, ‘days’, ‘want’, ‘worry’,* and *‘infectious’,* which concerned patients’ personal experiences of infection, quarantine, and treatment. In contrast, words such as ‘*Wuhan’*, ‘*life’*, ‘*world’*, and ‘*patient’* were used more frequently in health controls compared to the patient group, suggesting that healthy controls were more concerned with external circumstances such as the community and the country rather than themselves.”

**Table 3.** Rationale of Log-likelihood ratio (LL) for word identity comparison [34-36]

|  | Patient  Narratives (i*) | Control  Narratives (i*) | Total |
| --- | --- | --- | --- |
| Frequency of Target Word (O*) | a | b | a+b |
| Frequency of other words | c-a | d-b | c+d-a-b |
| Total (*N) | c | d | c+d |

$$-2\ln\lambda=2\sum_{i} O_{i} \ln\left( \frac{O_{i}}{E_{i}} \right), where E_{i}=\frac{N_{i}\sum_{i} O_{i}}{\sum_{i} N_{i}}$$

**Table 4.** Most distinctive words of SAD narratives between the two matched groups

| Word Feature | Log- likelihood | Patient (N=34) | | Control (N=24) | | Over/ Underuse |
| --- | --- | --- | --- | --- | --- | --- |
|  |  | **Frequency** | **Relative Frequency** | **Frequency** | **Relative Frequency** |  |
| *then* | 52.53 | 178 | 0.63% | 226 | 1.30% | - |
| *not* | 48.14 | 609 | 2.16% | 224 | 1.29% | + |
| *Wuhan* | 46.30 | 11 | 0.04% | 48 | 0.28% | - |
| *anyway* | 43.98 | 70 | 0.25% | 4 | 0.02% | + |
| *possibly* | 42.66 | 79 | 0.28% | 123 | 0.71% | - |
| *isolation* | 41.81 | 80 | 0.28% | 7 | 0.04% | + |
| *no* | 39.47 | 160 | 0.57% | 34 | 0.20% | + |
| *for sure* | 32.94 | 61 | 0.22% | 5 | 0.03% | + |
| *days* | 29.95 | 130 | 0.46% | 29 | 0.17% | + |
| *want* | 28.81 | 192 | 0.68% | 55 | 0.32% | + |
| *life* | 27.14 | 9 | 0.03% | 32 | 0.18% | - |
| *sister* | 25.99 | 27 | 0.10% | 0 | 0.00% | + |
| *world* | 25.98 | 1 | 0.00% | 17 | 0.10% | - |
| *patient* | 25.64 | 7 | 0.02% | 28 | 0.16% | - |
| *worry* | 25.57 | 34 | 0.12% | 1 | 0.01% | + |
| *infectious* | 25.02 | 26 | 0.09% | 0 | 0.00% | + |

Note: LL values of 15.13 or higher are significant (d.f.=1, p<0.0001)

References

21. Hong, K.; Nenkova, A.; March, M. E.; Parker, A. P.; Verma, R.; Kohler, C. G. Lexical use in emotional autobiographical narratives of persons with schizophrenia and healthy controls. Psychiatry Research 2015, 225(1-2),40-49.
30. Xu, L.; Lin, H.; Pan, Y.; Ren, H.; Chen, J. Constructing the affective lexicon ontology. Journal of the China Society for Scientific and Technical Information 2008, 27(2), 180-185.
32. Che, W.; Feng, Y.; Qin, L.; Liu, T. N-LTP: An open-source neural language technology platform for Chinese. Available online: https://arxiv.org/abs/2009.11616 (accessed on 27 February 2022).

33. Bird, S; Klein, E; Loper, E. *Natural Language Processing with Python*. O’Reilly Media Inc, 2009. <https://www.nltk.org/book/>

34.	Williams, K. The failure of Pearson's goodness of fit statistic. Journal of the Royal Statistical Society. 1976, 25(1) 49.

35.	Rayson, P. E. Matrix: A statistical method and software tool for linguistic analysis through corpus comparison. Lancaster University, 2003.

36.	Rayson, P., Garside R. Comparing corpora using frequency profiling. In The Workshop on Comparing Corpora. 2000, 1-6.

**• Page 3 lines 146-149: It seems more like a result rather than a methodological description. Please check.**

**Response:** We have revised these lines on page 4, as copied below:

“…In the discourse that follows, the generic features of the emotional narratives (i.e., mean word-length, words per sentence, sentences per narrative, and words per narrative), frequency of emotional words, and significant word identity features between the two matched groups were computed quantitatively by the text-mining method. Based on text analysis of emotional narratives, differences between persons infected with COVID-19 and healthy controls were measured with respect to each of their mental health statuses during the COVID-19 pandemic.”

**• Lines 162-180: How were interview's domains defined? Please clarify.**

**Response:** we have defined the interview’s domains on page 4, as copied below:

“The interview domain concerned multiple dimensions of their lived experience during COVID-19, such as work and daily life, social isolation, family support, social relationships, attitude towards death, comments on medical service, and national policy [11-12, 22, 29].”

**• The rationale of choosing two data collection periods (i.e., Summer and Autumn-Winter) should be clarified. Did the authors check data for differences across the two periods? I suggest discussing this issue further.**

**Response:** we clarified the reasons for choosing two data collection periods on page 5:

“The interviews were arranged during summer and autumn-winter as dictated by participants’ requirement and their pandemic situation.”

We also mentioned it in the *Limitations and future research* section on page 17, which reads as:

“Furthermore, the interviews were conducted during two different waves of the COVID-19 pandemic within China, namely summer and autumn-winter, across 2020-2021. The dynamic change of emotional status among the two groups across the two periods may have long-term effects”

…”Enquires along this line can control the intervals of different interviews and compare emotional symptoms of COVID-19 patients across different periods…”

**• In Table 2, what do the initials in brackets mean? Please add relevant footnotes.**

Response: we have added a note below Table 2:

“Note: The initials in brackets are the original ID tags in Xu et al.,’s [30] emotion dictionary. P stands for positive and N for negative.”

**• Some details mentioned at the beginning of the Results section need to be disclosed in the Materials and Methods section. Please revise the logical flow of the Methods section to include all relevant methodological details in order to let the reader fully understand the analyses.**

**Response:** Sorry for overlooking this specification. We have added a paragraph in the “Measures” of the Materials and Methods section on page 7 to specify the generic features of emotional narratives at the beginning of the Results section, as copied below:

*Analysis of generic features of emotional narratives*

“In the present study, generic features of emotional narratives, including Type-Token ratio, mean word-length, average number of words per sentence, number of sentences per narrative, and number of words per narrative were measured among patients and healthy controls. The Type-Token ratio shows the number of unique words divided by the total number of words in the emotional narratives corpus. A lower ratio indicates more use of the same words in the text. Mean word-length refers to the average number of Chinese characters per word. Longer words are usually more complex and indicates better use of language. Regarding average number of words per sentence, longer sentences tend to be syntactically more complex and express more complex ideas. As for number of sentences per narrative, more sentences in a specific emotional narrative of similar length indicate the use of shorter, less complex sentences. Total number of words per narrative specifies word frequency in a specific emotional narrative [21].”

Reference

22. Hong, K.; Nenkova, A.; March, M. E.; Parker, A. P.; Verma, R.; Kohler, C. G. Lexical use in emotional autobiographical narratives of persons with schizophrenia and healthy controls. Psychiatry Research 2015, 225(1-2),40-49.

**• Moreover, the techniques to implement sentiment analyses are not clearly presented in the Methods. Please revise related sentences, also referring to commonly used approaches such as the Valence Aware Dictionary for sEntiment Reasoning (VADER) and the Bidirectional Encoder Representations from Transformers (BERT) (** [**https://doi.org/10.1192/j.eurpsy.2021.3**](https://doi.org/10.1192/j.eurpsy.2021.3) **)**

**Response:** Sorry for not using VADER and BERT**.** The present study used NLTK (Natural Language Toolkit), an open source Python library for natural language processing [33], to conduct sentiment analysis of the interview texts. VADER is perfect for English sentiment analysis. As our text was original Chinese, we utilized Chinese Affective Lexicon Ontology [30], which includes seven basic traditional Chinese emotions called qi qing七情 (i.e., fear 惧, anger 怒, joy 乐, sadness 哀, disgust 恶, surprise 惊, and good 好), and 21 emotional subcategories over 27,466 lexical entries. This Chinese Affective Lexicon ontology has widely been used in the sentiment analyses of Chinese Texts.

We have added a whole “Measures” section in the Methods to report the techniques to implement sentiment analyses. Take the analysis of emotional words on pages 7-8 for example:

***Analysis of frequency of emotional words***

“To compute sentiment statistics, we used the package called ‘*CategorizedCorpusReader.words’ (categories=’group’)* in NLTK to calculate frequency of emotional words from specific group of interest (e.g., patient or control, positive or negative, HAPPY or SAD). First, words with no substantive meaning (functional words, punctuations) were filtered out, based on the stopwords list of Harbin Institute of Technology [32]. Next, frequency of emotional polarity and 5/21 categories of emotional words were extracted according to the Chinese Affective Lexicon ontology [30], by utilizing *‘probability.ConditionalFreqDist’* class in NLTK. By doing so, a conditional frequency distribution table was generated. *ConditionalFreqDist* (CFD) is essentially word frequency data stored in *key:value* pairs, where ‘*key’* is the user-defined condition and ‘*value’* is word frequency distribution (a *‘FreqDist’* class) of a group satisfying the condition. CFD is able to create individual ‘*FreqDist*’ objects with specific conditions of various sentiments (i.e., polarity, primary, or subcategorized emotions) and source of observation (i.e., patient or control).

Utilizing these tools, we conducted sentiment analysis by selecting the frequency of emotional vocabulary for each group according to different conditions. For instance, the occurrence of the vocabulary group of the primary emotion ‘*DISGUST’* among healthy controls was retrieved with the query (*CFD['control']['disgust']*). Other emotional types were measured in a similar way (e.g., *CFD [‘patient’][‘NC’]* calculated the frequency of fear related words among the patient group). Apart from searching for individual observation values, we used the plot function in *FreqDist* and the Python library, ‘*Matplotlob’,* to visualize the frequency of different types of emotional words by groups.”

See more measures in the previous comments regarding **subparagraph Measures.**

References

31. Xu, L.; Lin, H.; Pan, Y.; Ren, H.; Chen, J. Constructing the affective lexicon ontology. Journal of the China Society for Scientific and Technical Information 2008, 27(2), 180-185.
33. Che, W.; Feng, Y.; Qin, L.; Liu, T. N-LTP: An open-source neural language technology platform for Chinese. Available online: https://arxiv.org/abs/2009.11616 (accessed on 27 February 2022).

33. Bird, S; Klein, E; Loper, E. *Natural Language Processing with Python*. O’Reilly Media Inc, 2009. <https://www.nltk.org/book/>

**• There is some ambiguity between the two paragraphs of the Results section. For example lines 274-284 seem to fit best the next heading (Sentiment Frequency of emotional words and word identity). Please clarify.
 I suggest providing a clearer definition of the measures in the Materials and Methods section (see my previous comment about an additional “Measures” paragraph) and then following an unequivocal logical flow in the Results section according to what is described in the Methods. Moreover, types of emotional words (see Figure 4 footnotes) should be described in the Materials and Methods section.**

**Response:** As illustrated in previous comments about additional “Measures” paragraphs, we have added a whole “Measures” section on pages 7-9, which consists of three subsections regarding the measurement of *Generic Features of Emotional Narratives, Frequency of Emotional Words, and Significant Word Identity Features of Emotional Narratives*, in order to clarify the logical flow of the research findings in the Result.

On page 10, Table 6 (*The average number of words per narrative across five emotions*) and Figure 1 (*The average number of words per narratives across five emotions*) actually both measure the length of different types of emotional narratives, thus belong to the “words/narrative” in the generic features of emotional narratives in Table 5. Therefore, we keep them in the first section of Results. According to the logical flow of the newly added “Measures” section, Results section was subsequently divided into three sections, namely *Generic features of emotional narratives, Frequency of emotional words,* and *Significant word identity features of emotional narratives*.

Types of emotional words (see Figure 4 footnotes) were described in the *Emotional narrative tagging* section of Method on page 5, as copied below：

“…Based on Ekman’s six basic emotions, Xu et al., [30] established the Chinese Affective Lexicon Ontology, which includes seven basic traditional Chinese emotions called qi qing七情 (i.e., fear 惧, anger 怒, joy 乐, sadness 哀, disgust 恶, surprise 惊, and good 好), and 21 emotional subcategories over 27,466 lexical entries. The 21 emotional subcategories have largely extended the range of emotional experience analysis. To minimalize the basic emotions and enlarge the subcategories of emotions, the present study combined the five primary emotions of Hong et al., [21] and the 21 emotional subcategories of Xu et al., [30] in emotional narrative tagging (Table 2)…”

References

21. Hong, K.; Nenkova, A.; March, M. E.; Parker, A. P.; Verma, R.; Kohler, C. G. Lexical use in emotional autobiographical narratives of persons with schizophrenia and healthy controls. Psychiatry Research 2015, 225(1-2),40-49.
30. Xu, L.; Lin, H.; Pan, Y.; Ren, H.; Chen, J. Constructing the affective lexicon ontology. Journal of the China Society for Scientific and Technical Information 2008, 27(2), 180-185.

• **When interpreting Table 4, the authors focused on some emotions according to statistically significance (0.05 threshold). However, table 4 seems to show an additional perspective about potential emotional differences between patients and controls. In particular, although some estimates were not statistically significant, it shows that, as a whole, controls were likely to express different emotions through a higher number of words as compared with patients, except for anger. Although these results should be interpreted with caution, this perspective should be taken into account. I suggest the authors to revise relevant sentences in the Results section and add a comment on that in the Discussion section. Do you think that this might be related to the time interval between COVID-19 infection and the interview? In other words, can this be considered a short- or also a long-term effect? What are potential explanations? Please add a comment in the Discussion section, also considering available information from collected data.**

**Response:** The reviewer is right that there is more interesting results apart from the statistically significant measurements about the length of emotional narratives. We definitely take this point into account in both Results and Discussion sections.

In the Result section, we added several sentences on page 9, as copied below:

“…It is noteworthy that although narratives of anger and disgust were not statistically significant, as a whole, healthy controls were likely to express different emotions through a higher number of words as compared with patients, except for narratives of anger.”

In the Discussion section, we have added the detailed explanations about the surprising results regarding the narratives of anger on page 15, as copied below:

“…A surprising finding in the present study is that narratives of anger were longer in COVID-19 patients than in healthy controls, which was in sharp contrast to the narratives of fear, happiness, sadness, and disgust. One explanation for this finding is that the long period of quarantine and relative lack of social support led to severe symptoms of worry, anxiety, depression, anger, and irritation among COVID-19 patients, given the perception of social isolation [11]. In our interviews, most patients were infected by SARS-CoV-2 passively by their colleagues, neighbors, or family members. They denied the fact that they were infected and tended to attribute the infection to other people. As a result, the salient ‘angry’ emotion emerged in the minds of COVID-19 patients during the first stage of infection [17]. Furthermore, when COVID-19 patients experienced social stigmatization and extreme loneliness, it is possible that their anger would evolve into an extreme emotional disorder.”

References

11. Deng, Y.; Wang, L.; Yang, J.; Xie, L.; Chen, Y. How covid-19 patient narratives concerning reinfection mirror their mental health: A case series. Psychiatria Danubina 2021, 33(1), 114-119.

17. Sun, N.; Wei, L.; Wang, H.; Wang, X.; Gao, M.; Hu, X.; Shi, S. Qualitative study of the psychological experience of COVID-19 patients during hospitalization. Journal of Affective Disorders 2021, 278, 15-22.

**• Furthermore, the authors should clarify whether they collected meta data such as information on therapy/recovery, but also gender, age, etc… or they should acknowledge the lack of key information as a study limitation. Individual-level correlates may play a role that needs to be taken into account for future research. Indeed, based on available evidence, different individual-level, socio-demographic and pre-existing clinical correlates are likely to be associated with an increased mental health burden and should be taken into account** ( <https://doi.org/10.1016/S0140-6736(20)30460-8> ; <https://doi.org/10.1016/j.neubiorev.2021.10.010> ; <https://doi.org/10.1016/S0140-6736(21)02143-7> )

**Response:** The reviewer is right that individual-level correlates may impact the mental health burden of people during COVID-19. We have acknowledged the lack of key information as a study limitation and propose future direction on pages 16-17, as copied below:

“This study has several limitations……Third, we investigated the emotional disorders of COVID-19 patients under collective emotions as opposed to healthy controls. Individual-level correlates, such as clinical therapy, quarantine period, education level, occupation, and income, were therefore underestimated and not presented in our data. “

“In light of these challenges……Finally, future sentiment analysis of COVID-19 patients can consider different individual-level, socio-demographic, and pre-existing clinical correlates, given that these factors have the potential to be used as markers to predict different emotional and mental burdens among different categories of patients [3, 43-44].”

References

3. Santomauro, Damian F et al. Global prevalence and burden of depressive and anxiety disorders in 204 countries and territories in 2020 due to the COVID-19 pandemic. The Lancet, 398, 10312, 1700- 1712. https://doi.org/10.1016/S0140-6736(21)02143-7
43. Crocamo C, Bachi B, Calabrese A, Callovini T, Cavaleri D, Cioni RM, Moretti F, Bartoli F, Carrà G. Some of us are most at risk: Systematic review and meta-analysis of correlates of depressive symptoms among healthcare workers during the SARS-CoV-2 outbreak. Neurosci Biobehav Rev. 2021b;131:912-922. doi: 10.1016/j.neubiorev.2021.10.010.
44. Brooks S. K.; Webster R K, Smith L E , et al. The psychological impact of quarantine and how to reduce it: rapid review of the evidence. The Lancet, 2020, 395(10227):912-920. <https://doi.org/10.1016/S0140-6736(20)30460-8>

**• Lines 286-287: “We focused exclusively on examining lexical features in the emotional narratives related to the emotionality.” Please clarify. In addition, did the authors mean frequency distribution or rather sentiment polarity?**

**Response:** Sorry for the vague depiction. We have clarified this description on page 11, as copied below:

**“Frequency of emotional words”**

“We focused on examining frequency distribution of emotional words related to sentiment polarity and the 5/21 emotional classification. The frequency distribution of emotional words between the two matched groups is shown in Figure 2, Figure 3, and Figure 4….”

**• Qualitative results are not adequately justified through representative excerpts from the data. Therefore, some excerpts from the qualitative analysis should be provided and discussed in order to let the reader better understand the results, along with those provided next to Table 2.**

Response: We have added examples and qualitative analysis in the last section of Results on page 14, as copied below:

“It is worth noting that patient narratives of happiness encompass words belonging to the ‘fear’ and ‘disgust’ domains. The reason is that word identity feature was extracted from a bag-of-words model, as shown in the following examples:

“++HAPPY(PE)++I don’t think I showed fear of death. I think death is not dreadful. We have elderly family members, so I don’t think death is dreadful. We can keep a positive view about life and treat death a natural thing. I think everyone will die, and will finally reach the end of life. This is a natural process. For me, death is not dreadful. (Patient 13)”

“++HAPPY(PE)++ I never felt irritable in mood; I felt peaceful during hospital treatment. At the beginning of hospital isolation, I showed severe symptoms and event felt difficult to breathe and to eat. However, three days later I felt like eating an ox, and I could eat meals of two persons every day. I felt happy with good appetite and my immunity enhanced. I recovered very soon. (Patient 27)”

“In the preceding two excerpts, Patient 13 and Patient 27 described their positive emotion toward COVID-19 infection. They demonstrated no fear toward death, and felt ‘peaceful’ with the virus. These two narratives of happiness encompasses negative words in the domain of fear and disgust, such as ’fear’, ’dreadful’ ,’die’, ’irritable’, ’severe symptoms’, and ’difficult’. However, they are syntagmatically combined with words of negation and represent patients’ positive emotions concerning their confidence in recovery.”

Also see the point following the next one.

**• The Discussion section appears to jump to conclusions. These need to be clarified and justified. For example lines 351-352; 356-358 (Sentiment or content analysis?); 362-364; 371-373. Please give additional explanations and consider providing adequate references. As a result, the first paragraph of the Discussion needs to be extensively revised.**

**Response:** we have extensively revised the first paragraph of the Discussion by providing relevant references in this field, and clarified our findings on pages 14-15, as copied below:

“Research into mental and physical health relating to the COVID-19 pandemic has applied text analysis to social media data such as Twitter, Facebook, and Reddit to forecast the possible emergence of emotional disorders and mental illness [23-26]. Emotional narratives of patients with confirmed COVID-19 disease has proven to be an important marker to gain a deeper understanding of emotion-related mental health issues due to COVID-19 [22]. Drawing on the text mining approach, this study aimed to examine differences in emotional status between matched groups of COVID-19 patients and healthy controls, who narrated their lived experiences of COVID-19. As part of our established procedure to assess evoked emotional discourse, we tagged narratives of happy, sad, angry, fearful, and disgusted emotions of both groups. Automated analysis of generic features, sentiment word frequency, and significant word features in the emotional narratives between COVID-19 patients and healthy controls reflected their prevailing mental health status. As for generic features of emotional narratives, we observed that persons afflicted by COVID-19 employed significantly more words per sentence, had significantly fewer sentences per narrative, and used significantly fewer words per narrative, as compared to healthy controls. Patients demonstrated generally lower complexity of emotional language across different types of emotional narratives as opposed to healthy controls, other than anger. It is noteworthy that patients’ emotional narratives of fear, happiness, and sadness were significantly shorter compared to healthy controls. Regarding different categories of emotional words, patients generally used more emotional words, in particular, more words of fear (i.e., dread and depression), disgust (i.e., criticism), and happiness (i.e., joy, trust, and like), as compared to healthy controls. This suggests that patients’ emotional symptoms at the lexical level were concentrated on the emotions of fear and disgust. The analysis of the significant word features of emotional narratives showed that COVID-19 patients talked more about themselves or their family surviving COVID-19 in terms of unexpected infection, isolation, and macro-identity [22], while healthy controls mainly talked about others. “

We have also give additional explanations for the 2nd paragraph of Discussion on page 15, as copied below:

“At the sentence level, patients used longer sentences to describe their emotion, revealing that their attention to topic was not as fixed as that of healthy controls, and that their emotions were unstable. This was associated with their emotional perturbation due to the COVID-19 infection. At the narrative level, healthy controls demonstrated generally higher complexity and more expressive emotional language (i.e., narratives of fear, sadness, happiness, and disgust) than COVID-19 patients, suggesting that patients’ mental health disruptions had limited the linguistic range of expression of their emotional experiences. This research finding echoes the observations of prior psychiatric studies (e.g., of schizophrenia) concerning abnormalities in the production of coherent discourse due to cognitive dysfunction involving attention and thoughts [21]. A surprising finding in the present study is that narratives of anger were longer in COVID-19 patients than in healthy controls, which was in sharp contrast to the narratives of fear, happiness, sadness, and disgust. One explanation for this finding is that the long period of quarantine and relative lack of social support led to severe symptoms of worry, anxiety, depression, anger, and irritation among COVID-19 patients, given the perception of social isolation [11]. In our interviews, most patients were infected by SARS-CoV-2 passively by their colleagues, neighbors, or family members. They denied the fact that they were infected and tended to attribute the infection to other people. As a result, the salient ‘angry’ emotion emerged in the minds of COVID-19 patients during the first stage of infection [17]. Furthermore, when COVID-19 patients experienced social stigmatization and extreme loneliness, it is possible that their anger would evolve into an extreme emotional disorder. Overall, our results are consistent with those of published literature, which shows that COVID-19 patients are particularly vulnerable to the emotional impact of coronavirus [11-14, 16, 19-20, 22, 29].”

Key References

11. Deng, Y.; Wang, L.; Yang, J.; Xie, L.; Chen, Y. How covid-19 patient narratives concerning reinfection mirror their mental health: A case series. Psychiatria Danubina 2021, 33(1), 114-119.
17. Sun, N.; Wei, L.; Wang, H.; Wang, X.; Gao, M.; Hu, X.; Shi, S. Qualitative study of the psychological experience of COVID-19 patients during hospitalization. Journal of Affective Disorders 2021, 278, 15-22.

21. Hong, K.; Nenkova, A.; March, M. E.; Parker, A. P.; Verma, R.; Kohler, C. G. Lexical use in emotional autobiographical narratives of persons with schizophrenia and healthy controls. Psychiatry Research 2015, 225(1-2),40-49.

23. Deng, Y.; Li, H.; Park, M. Emotional Experiences of COVID-19 Patients in China: A Qualitative Study. Int. J. Environ. Res. Public Health 2022, 19, 9491. <https://doi.org/10.3390/ijerph19159491>
24. Crocamo C, Viviani M, Famiglini L, Bartoli F, Pasi G, Carrà G (2021). Surveilling COVID-19 Emotional Contagion on Twitter by Sentiment Analysis. European Psychiatry, 64(1), e17, 1–6. <https://doi.org/10.1192/j.eurpsy.2021.3>
25. Low DM, Rumker L, Talkar T, Torous J, Cecchi G, Ghosh SS. Natural Language Processing Reveals Vulnerable Mental Health Support Groups and Heightened Health Anxiety on Reddit During COVID-19: Observational Study. J Med Internet Res. 2020 Oct 12;22(10):e22635. doi: 10.2196/22635.
26. Patel R, Smeraldi F, Abdollahyan M, Irving J, Bessant C. Analysis of mental and physical disorders associated with COVID-19 in online health forums: a natural language processing study. BMJ Open. 2021 Nov 5; 11(11): e056601. doi: 10.1136/bmjopen-2021-056601.
27. Viviani M, Crocamo C, Mazzola M, Bartoli F, Carrà G, Pasi G. Assessing vulnerability to psychological distress during the COVID-19 pandemic through the analysis of microblogging content. Future Gener Comput Syst. 2021 Dec;125:446-459. doi: 10.1016/j.future.2021.06.044.

**• How was happiness combined with emotional words belonging to fear and disgust domains? In other words, happiness is somehow opposite to fear and disgust. How did the authors interpret these findings? (Consistently, please report the name of the emotion.)**

**Response**: Sorry for the confusing presentation of the results. The word identity features occur in the larger unit of emotional narratives in a wide variety of contexts. This is a possible consequence of using a simple bag-of-words model to compute Log-likelihood ratio. In Table 7, we have clarified the five basic emotional narratives under which the bag-of-words were extracted. In this regard, word identity feature is interpreted in a bag-of-words across the five basic emotional narratives. We have added several paragraphs to clarify this issue with typical examples to illustrate how negative words occur in the narratives of happiness on pages 14, as copied below:

“It is worth noting that patient narratives of happiness encompass words belonging to the ‘fear’ and ‘disgust’ domains. The reason is that word identity feature was extracted from a bag-of-words model, as shown in the following examples:

“++HAPPY(PE)++I don’t think I showed fear of death. I think death is not dreadful. We have elderly family members, so I don’t think death is dreadful. We can keep a positive view about life and treat death a natural thing. I think everyone will die, and will finally reach the end of life. This is a natural process. For me, death is not dreadful. (Patient 13)”

“++HAPPY(PE)++ I never felt irritable in mood; I felt peaceful during hospital treatment. At the beginning of hospital isolation, I showed severe symptoms and event felt difficult to breathe and to eat. However, three days later I felt like eating an ox, and I could eat meals of two persons every day. I felt happy with good appetite and my immunity enhanced. I recovered very soon. (Patient 27)”

“In the preceding two excerpts, Patient 13 and Patient 27 described their positive emotion toward COVID-19 infection. They demonstrated no fear toward death, and felt ‘peaceful’ with the virus. These two narratives of happiness encompasses negative words in the domain of fear and disgust, such as ’fear’, ’dreadful’ ,’die’, ’irritable’, ’severe symptoms’, and ’difficult’. However, they are syntagmatically combined with words of negation and represent patients’ positive emotions concerning their confidence in recovery.”

**• Furthermore, based on available evidence, the authors should add a comment in the Discussion section on the use of novel machine learning algorithms and relevant methods grounded on natural language processing techniques as a promising approach for the design of a critical surveillance system to manage pandemic-like scenarios. These considerations should contribute to emphasize the importance of an emotional discourse analysis that may possibly benefit also from social media integration for timely preventive interventions (** [**https://doi.org/10.1192/j.eurpsy.2021.3**](https://doi.org/10.1192/j.eurpsy.2021.3) **)**

**Response:** Thanks for this constructive comment. We have added a paragraph at the end of Discussion section on page 16, as copied below:

“In the era of big data, emotional discourse analysis of COVID-19 patient data based on novel machine learning algorithms, and recent developments based on natural language processing techniques are promising approaches for the design of a critical surveillance system to manage pandemic-like health scenarios. To assess the emotional status of COVID-19 patients in a comprehensive manner, large-scale emotional discourse analysis based on interviews and stored social media data can be integrated into essential surveillance tools for the management of pandemic-related mental health issues. These measures represent a novel prediction and preventive approach to effectively mitigate emotional disorders resulting from COVID-19, and also allow timely preventive interventions to be implemented for COVID-19 patients [22, 43].”

References

22. Deng, Y.; Li, H.; Park, M. Emotional Experiences of COVID-19 Patients in China: A Qualitative Study. Int. J. Environ. Res. Public Health 2022, 19, 9491. <https://doi.org/10.3390/ijerph19159491>
43. Crocamo C, Bachi B, Calabrese A, Callovini T, Cavaleri D, Cioni RM, Moretti F, Bartoli F, Carrà G. Some of us are most at risk: Systematic review and meta-analysis of correlates of depressive symptoms among healthcare workers during the SARS-CoV-2 outbreak. Neurosci Biobehav Rev. 2021b;131:912-922. doi: 10.1016/j.neubiorev.2021.10.010.

• **I suggest revising the Conclusion paragraph, by making the first part more compact in order to let the reader better understand the take home message.**

**Response:** we have revised the Conclusion paragraph in accordance with the reviewer’s suggestion and also added several lines to emphasize the important role real-world data play in mental health surveillance on page 17, as copied below:

“Our text-mining-based emotional discourse analysis focused on generic features, emotional word frequency, and significant word identity features of emotional narratives in COVID-19 patients and healthy controls. Our results indicate generally lower complexity and less expressive emotional narratives in COVID-19 patients than in healthy controls. This reveals that psychological disruptions appear to inhibit the production of a detailed and coherent emotional discourse in COVID-19 patients. At the lexical level, COVID-19 patients used more words of fear, disgust, and happiness, as compared to healthy controls. Furthermore, patients narrated more words with respect to self or family while healthy controls talked mainly about others. Overall, while the emotional symptoms of COVID-19 patients at the lexical level focused on the negative emotions of fear and disgust concerning their confirmed infection and long period of treatment, their emotion was positively regulated by their recovery and adequate support from medical staff [22].”

“…During the present COVID-19 era, large-scale text mining of real-world data, such as interview data, social media data, and online health forum data, reveals the possibility of the design of a central surveillance system to manage emotional and mental health issues concerning COVID-19 (and possibly other future pandemic-like) infection. This system can be harnessed to inform and guide public healthcare policy and provide useful insights into developing novel therapeutics related to mental health symptoms [25].”

References

22. Deng, Y.; Li, H.; Park, M. Emotional Experiences of COVID-19 Patients in China: A Qualitative Study. Int. J. Environ. Res. Public Health 2022, 19, 9491. <https://doi.org/10.3390/ijerph19159491>
25. Patel R, Smeraldi F, Abdollahyan M, Irving J, Bessant C. Analysis of mental and physical disorders associated with COVID-19 in online health forums: a natural language processing study. BMJ Open. 2021 Nov 5; 11(11): e056601. doi: 10.1136/bmjopen-2021-056601.

**Reviewer #2**:

In the study presented in this article, the authors used semi-structured interviews and affirm to use natural language processing to analyze the characteristics of lived experience narratives between patients with COVID-19 and a group of healthy patients related to five basic emotions. The aim of the work was to identify differences in emotional state between the two groups of participants. The results obtained indicate generally higher complexity and more expressive emotional language in the healthy patient group than in the COVID-19 patients.

The work has a rather relevant objective but, in my opinion, it fails to be convincing with respect to the results obtained, this due to several weaknesses and naive solutions used in the interpretation of the results.

 **- First of all, semi-structured telephone interviews were used in the study. It is not discussed sufficiently how it is ensured that the interview modality and the questions asked do not in any way imply bias with respect to the answers provided. Has it been thought to also consider other content generated by these users independently of the telephone interview? There are works that have tried to assess the psychological vulnerability of users with respect to this aspect, and other works more similar to the proposed work, which were not mentioned in the work**, for example:

* Low, Daniel M., et al. "Natural language processing reveals vulnerable mental health support groups and heightened health anxiety on reddit during covid-19: Observational study." Journal of medical Internet research 22.10 (2020): e22635.
* Patel, Rashmi, et al. "Analysis of mental and physical disorders associated with COVID-19 in online health forums: a natural language processing study." BMJ open 11.11 (2021): e056601.
* Viviani, Marco, et al. "Assessing vulnerability to psychological distress during the COVID-19 pandemic through the analysis of microblogging content." Future Generation Computer Systems 125 (2021): 446-459.

**It is true that the authors take into consideration specific groups of users affected and not affected by COVID-19, however there are interesting aspects of the previous works that could be taken into consideration, the lack of which constitutes another weak point of the presented work.**

**Response:** Thanks for this constructive comment. We have added two paragraphs in the Introduction section, reporting literature regarding the COVID-19 related text analysis and mental health, and identifying the research gap on pages 2-3, as copied below:

“…Recent studies have used natural language processing (NLP) techniques to extract real-world text data concerning COVID-19 from online resources such as Twitter, Facebook, and Reddit, in order to provide automated population-level health surveillance [23-26]. For instance, Crocamo et al., [23] conducted sentiment analysis of 3,308,476 English tweets concerning COVID-19 discussions between January 19 and March 3, 2020, by computing a polarity compound score and using a transformer-based model. Their results demonstrated an increasing trend towards negative sentiment as the COVID-19 pandemic proceeded. Low et al., [24] used NLP techniques to detect changes in mental health support groups (e.g., Schizophrenia, Suicide Watch, Depression) and non-mental health groups (e.g., Personal Finance, Conspiracy Theory) at the outbreak of COVID-19. Drawing on the Reddit data of 826,961 users by using unsupervised methods such as topic modeling and clustering, Low et al., [24] found that support groups related to attention-deficit/hyperactivity disorder, eating disorders, and anxiety showed the most negative semantic changes during COVID-19. Health anxiety was found to be a salient theme across Reddit through machine learning analyses. Patel et al., [25] used data mining methods to compute 739,434 COVID-19 related posts by 53,134 users from online health forums such as Health Boards and Inspire & Health Unlocked from 1 January 2020 to 31 May 2020. Their results showed that the global lockdown policy had led to an increase of discussion related to COVID-19-related disorders, and 25% of the COVID-19-related titles mentioned an associated physical or mental health comorbidity. Viviani et al., [26] conducted word identity and sentiment analysis of vulnerability to psychological distress in the COVID-19 context by gathering texts related to COVID-19 on the Twitter microblogging platform between August and December 2020. The automatic analysis indicated that the social distancing scenario was associated with ‘annoyance’ and ‘protest’, while the vaccine scenario was linked to ‘killing’ and ‘murder’. Regarding anxiety, the target scenarios of social distancing and symptoms & hospitalization were closely related to risk, worry, and fear. As for vulnerability scores in the word categories, the lexicon identifiers regarding greater psychological vulnerability fell into the ‘anger’ and ‘risk’ categories, in particular for social distancing and vaccines & vaccinations. There was a large increase in vulnerability identifiers in the ‘death’ category shared by the ‘symptoms & hospitalization’ scenario. “

“Text mining based on real-world data is a sensitive method to reveal emotional and mental health burden during COVID-19, given that it provides an effective marker to identify the mental status of vulnerable groups and alarming themes during the ongoing pandemic [24]. It is noteworthy that the automatic analysis of COVID-19 patients’ texts is somehow overlooked in the literature. Along this line of enquiry, the present study combined in-depth semi-structured interviews with the text-mining method to investigate features in lived experience narratives regarding COVID-19 in persons with COVID-19 and in healthy controls related to five basic emotions….”

References

23. Crocamo C, Viviani M, Famiglini L, Bartoli F, Pasi G, Carrà G (2021). Surveilling COVID-19 Emotional Contagion on Twitter by Sentiment Analysis. European Psychiatry, 64(1), e17, 1–6. <https://doi.org/10.1192/j.eurpsy.2021.3>
24. Low DM, Rumker L, Talkar T, Torous J, Cecchi G, Ghosh SS. Natural Language Processing Reveals Vulnerable Mental Health Support Groups and Heightened Health Anxiety on Reddit During COVID-19: Observational Study. J Med Internet Res. 2020 Oct 12;22(10):e22635. doi: 10.2196/22635.
25. Patel R, Smeraldi F, Abdollahyan M, Irving J, Bessant C. Analysis of mental and physical disorders associated with COVID-19 in online health forums: a natural language processing study. BMJ Open. 2021 Nov 5; 11(11): e056601. doi: 10.1136/bmjopen-2021-056601.
26. Viviani M, Crocamo C, Mazzola M, Bartoli F, Carrà G, Pasi G. Assessing vulnerability to psychological distress during the COVID-19 pandemic through the analysis of microblogging content. Future Gener Comput Syst. 2021 Dec;125:446-459. doi: 10.1016/j.future.2021.06.044.

- **In fact, the authors claim to use "the natural language processing method". Regardless of the fact that the sentence itself is incorrect, as NLP includes a series of techniques for natural language processing (and it is not a "method"), the fact remains that the text analysis method used by the authors is rather simple and may in any case refer to techniques of "text mining" rather than "natural language processing". In fact, no semantic or natural language understanding analysis is carried out, as frequentist analyzes of the appearance of particular textual characteristics within the interviews are essentially taken into consideration.**

**Response:** The reviewer is right that the present study utilized the techniques of text mining to investigate features in emotional narratives in persons with COVID-19 and healthy controls. Hence, we have revised the title as “Emotional Discourse Analysis of COVID-19 Patients and their Mental Health: A Text Mining Study”. We also replaced the NLP description with “text mining” throughout the paper.

Furthermore, in the newly added “Measures” section (suggested by reviewer 1), we have specified the techniques used for sentiment analysis, by drawing on NLTK (Natural Language Toolkit)—an open source Python library for natural language processing (<https://www.nltk.org/book/>) [33]. The specific measures were described in details on pages 7-9 as copied as below:

**“Measures”**

“Based on the preprocessed corpus labeled with emotional tags, quantitative analysis of the emotional narrative followed. The first step involved automated analysis of generic features of the emotional narratives of COVID-19 patients and healthy controls, including mean word-length, words per sentence, sentences per narrative, and words per narrative. In the second step, sentiment analysis was implemented by calculating the frequency of emotional words. In doing so, differences regarding polarity and intensity of emotions were exposed between patients and controls. Lastly, significant features of word identity across the emotional narratives were compared between the two matched groups. “

***Analysis of generic features of emotional narratives***

“In the present study, generic features of emotional narratives, including Type-Token ratio, mean word-length, average number of words per sentence, number of sentences per narrative, and number of words per narrative were measured among patients and healthy controls. The Type-Token ratio shows the number of unique words divided by the total number of words in the emotional narratives corpus. A lower ratio indicates more use of the same words in the text. Mean word-length refers to the average number of Chinese characters per word. Longer words are usually more complex and indicates better use of language. Regarding average number of words per sentence, longer sentences tend to be syntactically more complex and express more complex ideas. As for number of sentences per narrative, more sentences in a specific emotional narrative of similar length indicate the use of shorter, less complex sentences. Total number of words per narrative specifies word frequency in a specific emotional narrative [21].

The emotional narratives corpus was loaded into Python for generic feature analysis, drawing on the ‘*CorpusReader* ‘class in NLTK (Natural Language Toolkit)—an open source Python library for natural language processing [33]. This package made file opening and stream control robust, and lent support to large-scale text retrieving and operating of the current corpus. As *CorpusReader* class in NLTK provides various text processing measurements (e.g., *paras()*, *sents()*, *words()*), it was used to automatically measure grammatical categories, such as word, sentence, and paragraph, in specific narratives of patients and controls (with *fileids*, *categories* argument). In this regard, average X per Y = len(X) / len(Y) was computed with various sub-functions of *CorpusReader* classes including *reader.words()*, *reader.sents()*, *reader.paras()* in conditions such as *reader.words (categories=’patient’)*. ”

***Analysis of frequency of emotional words***

“To compute sentiment statistics, we used the package called ‘*CategorizedCorpusReader.words’ (categories=’group’)* in NLTK to calculate frequency of emotional words from specific group of interest (e.g., patient or control, positive or negative, HAPPY or SAD). First, words with no substantive meaning (functional words, punctuations) were filtered out, based on the stopwords list of Harbin Institute of Technology [32]. Next, frequency of emotional polarity and 5/21 categories of emotional words were extracted according to the Chinese Affective Lexicon ontology [30], by utilizing *‘probability.ConditionalFreqDist’* class in NLTK. By doing so, a conditional frequency distribution table was generated. *ConditionalFreqDist* (CFD) is essentially word frequency data stored in *key:value* pairs, where ‘*key’* is the user-defined condition and ‘*value’* is word frequency distribution (a *‘FreqDist’* class) of a group satisfying the condition. CFD is able to create individual ‘*FreqDist*’ objects with specific conditions of various sentiments (i.e., polarity, primary, or subcategorized emotions) and source of observation (i.e., patient or control).

Utilizing these tools, we conducted sentiment analysis by selecting the frequency of emotional vocabulary for each group according to different conditions. For instance, the occurrence of the vocabulary group of the primary emotion ‘*DISGUST’* among healthy controls was retrieved with the query (*CFD['control']['disgust']*). Other emotional types were measured in a similar way (e.g., *CFD [‘patient’][‘NC’]* calculated the frequency of fear related words among the patient group). Apart from searching for individual observation values, we used the plot function in *FreqDist* and the Python library, ‘*Matplotlob’,* to visualize the frequency of different types of emotional words by groups.”

***Analysis of significant word identity features of emotional narratives***

“Significant word identity features, such as the probabilistic tendency of specific words, can mark mental status of COVID-19 patients as compared to healthy controls. In this study, the Log-likelihood ratio (LL) was adopted to measure the difference in word identity between the two sub-corpora. Table 3 shows the rationales of the comparison and mathematical formula [34-36] that were implemented via ‘*AntConc’* software. Table 4 shows a comparison of word identity in SAD narratives between the two matched groups. Looking at ‘not’ and ‘no’ in Table 4, the high LL value suggests that patients narrated more negative experiences or attitudes compared to healthy controls. The same is true of words such as *‘isolation’, ‘days’, ‘want’, ‘worry’,* and *‘infectious’,* which concerned patients’ personal experiences of infection, quarantine, and treatment. In contrast, words such as ‘*Wuhan’*, ‘*life’*, ‘*world’*, and ‘*patient’* were used more frequently in health controls compared to the patient group, suggesting that healthy controls were more concerned with external circumstances such as the community and the country rather than themselves.”

**Table 3.** Rationale of Log-likelihood ratio (LL) for word identity comparison [34-36]

|  | Patient  Narratives (i*) | Control  Narratives (i*) | Total |
| --- | --- | --- | --- |
| Frequency of Target Word (O*) | a | b | a+b |
| Frequency of other words | c-a | d-b | c+d-a-b |
| Total (*N) | c | d | c+d |

$$-2\ln\lambda=2\sum_{i} O_{i} \ln\left( \frac{O_{i}}{E_{i}} \right), where E_{i}=\frac{N_{i}\sum_{i} O_{i}}{\sum_{i} N_{i}}$$

**Table 4.** Most distinctive words of SAD narratives between the two matched groups

| Word Feature | Log- likelihood | Patient (N=34) | | Control (N=24) | | Over/ Underuse |
| --- | --- | --- | --- | --- | --- | --- |
|  |  | **Frequency** | **Relative Frequency** | **Frequency** | **Relative Frequency** |  |
| *then* | 52.53 | 178 | 0.63% | 226 | 1.30% | - |
| *not* | 48.14 | 609 | 2.16% | 224 | 1.29% | + |
| *Wuhan* | 46.30 | 11 | 0.04% | 48 | 0.28% | - |
| *anyway* | 43.98 | 70 | 0.25% | 4 | 0.02% | + |
| *possibly* | 42.66 | 79 | 0.28% | 123 | 0.71% | - |
| *isolation* | 41.81 | 80 | 0.28% | 7 | 0.04% | + |
| *no* | 39.47 | 160 | 0.57% | 34 | 0.20% | + |
| *for sure* | 32.94 | 61 | 0.22% | 5 | 0.03% | + |
| *days* | 29.95 | 130 | 0.46% | 29 | 0.17% | + |
| *want* | 28.81 | 192 | 0.68% | 55 | 0.32% | + |
| *life* | 27.14 | 9 | 0.03% | 32 | 0.18% | - |
| *sister* | 25.99 | 27 | 0.10% | 0 | 0.00% | + |
| *world* | 25.98 | 1 | 0.00% | 17 | 0.10% | - |
| *patient* | 25.64 | 7 | 0.02% | 28 | 0.16% | - |
| *worry* | 25.57 | 34 | 0.12% | 1 | 0.01% | + |
| *infectious* | 25.02 | 26 | 0.09% | 0 | 0.00% | + |

Note: LL values of 15.13 or higher are significant (d.f.=1, p<0.0001)

In addition, we have added some qualitative analysis in the revised manuscripts to make up the limitations of lack of semantic or natural language understanding analysis. See page 14, as copied below:

“It is worth noting that patient narratives of happiness encompass words belonging to the ‘fear’ and ‘disgust’ domains. The reason is that word identity feature was extracted from a bag-of-words model, as shown in the following examples:

“++HAPPY(PE)++I don’t think I showed fear of death. I think death is not dreadful. We have elderly family members, so I don’t think death is dreadful. We can keep a positive view about life and treat death a natural thing. I think everyone will die, and will finally reach the end of life. This is a natural process. For me, death is not dreadful. (Patient 13)”

“++HAPPY(PE)++ I never felt irritable in mood; I felt peaceful during hospital treatment. At the beginning of hospital isolation, I showed severe symptoms and event felt difficult to breathe and to eat. However, three days later I felt like eating an ox, and I could eat meals of two persons every day. I felt happy with good appetite and my immunity enhanced. I recovered very soon. (Patient 27)”

“In the preceding two excerpts, Patient 13 and Patient 27 described their positive emotion toward COVID-19 infection. They demonstrated no fear toward death, and felt ‘peaceful’ with the virus. These two narratives of happiness encompasses negative words in the domain of fear and disgust, such as ’fear’, ’dreadful’ ,’die’, ’irritable’, ’severe symptoms’, and ’difficult’. However, they are syntagmatically combined with words of negation and represent patients’ positive emotions concerning their confidence in recovery.”

References

21. Hong, K.; Nenkova, A.; March, M. E.; Parker, A. P.; Verma, R.; Kohler, C. G. Lexical use in emotional autobiographical narratives of persons with schizophrenia and healthy controls. Psychiatry Research 2015, 225(1-2),40-49.
30. Xu, L.; Lin, H.; Pan, Y.; Ren, H.; Chen, J. Constructing the affective lexicon ontology. Journal of the China Society for Scientific and Technical Information 2008, 27(2), 180-185.
32. Che, W.; Feng, Y.; Qin, L.; Liu, T. N-LTP: An open-source neural language technology platform for Chinese. Available online: https://arxiv.org/abs/2009.11616 (accessed on 27 February 2022).
33. Bird, S; Klein, E; Loper, E. *Natural Language Processing with Python*. O’Reilly Media Inc, 2009. <https://www.nltk.org/book/>
34. Williams, K. The failure of Pearson's goodness of fit statistic. Journal of the Royal Statistical Society. 1976, 25(1) 49.
35. Rayson, P. E. Matrix: A statistical method and software tool for linguistic analysis through corpus comparison. Lancaster University, 2003.
36. Rayson, P., Garside R. Comparing corpora using frequency profiling. In The Workshop on Comparing Corpora. 2000, 1-6.

**- In evaluating users' emotions, the authors therefore follow an approach based essentially on the lexicon, without making any reference to recent developments in the NLP field which take into account both the semantics of words and the context in which these words appear (BERT, BioBERT, transformer-based approaches, use of deep neural networks).**

**Response:** As illustrated in the preceding comment, we have added a whole “Measures” section in the Method to specify our text mining approach in details. We are sorry for not using the recent NLP techniques such as BERT, BioBERT, and deep neural networks. We have acknowledged this weakness and pointed out the future direction in *Study limitations and future research* on pages 16-17 as copied below:

“This study has several limitations……A second limitation has to do with the techniques of sentiment analysis, which largely rested on the Chinese Affective Lexicon Ontology [30] and the NLTK (Natural Language Toolkit) in Python [33]. It should be noted that semantic analysis based on deep learning methods of NLP was underestimated… “

“In the light of these challenges……, Furthermore, recent developments in NLP techniques, such as transformer-based approaches (e.g., BERT, BioBert) and deep neural network learning can be utilized to conduct sentiment analysis of COVID-19 patient narratives. These models are able to measure both the semantics of emotional words and the context that these words occur in robustly….”

References

30. Xu, L.; Lin, H.; Pan, Y.; Ren, H.; Chen, J. Constructing the affective lexicon ontology. Journal of the China Society for Scientific and Technical Information 2008, 27(2), 180-185.
34. Bird, S; Klein, E; Loper, E. *Natural Language Processing with Python*. O’Reilly Media Inc, 2009. <https://www.nltk.org/book/>

**- Ultimately I can say that the work from the point of view of language analysis with respect to the problem in question is not particularly innovative compared to previous works of literature, and, in any case it refers to concepts that are ultimately not really used within the work (for example NLP).**
**Response:** The purpose of this study was to investigate psycholinguistic features in the emotional narratives of the Covid-19 patients, by employing NLTK (Natural Language Toolkit) in Python [33]. Compared with the literature, the gold of this research is that it may be the first study focusing on the text-mining of lived experience narratives of COVID-19 patients and their mental health status. It is true as the reviewer suggested that our study lacks the new NLP techniques. As illustrated in the preceding comment, we have acknowledged the limitation and pointed out the future direction regarding recent NLP techniques in *Study limitations and future research* on pages 16-17.

We thank the reviewer for pointing out this limitation directly. In our future research of COVID-19 patient texts, we will definitely make a sentiment classifier by adopting vectorized representation (e.g., word2vec) and nondeterministic models (e.g., BERT), or take a symbolic approach to develop a rule-based model (e.g., VADER).

References

33. Bird, S; Klein, E; Loper, E. *Natural Language Processing with Python*. O’Reilly Media Inc, 2009. <https://www.nltk.org/book/>

**I also believe that on a rather limited group of patients it is difficult to draw conclusions that are statistically relevant, even this aspect should have been discussed more carefully.**

**Response:** we acknowledged this weakness and proposed future direction in *Study limitations and future research* on page 17, as copied here:

“…This study has several limitations. First, we collected the narrative discourses of 34 COVID-19 patients and 24 healthy controls by convenience sampling. Our sample size was small and not entirely balanced, and our findings may thus not be generalizable to large populations.….”

“In the light of these challenges, we hope and anticipate that similar future investigations could draw on larger sample sizes of patient narratives……”

**Finally, I must say that the level of English should also be improved, because very often there are sentences that are not understandable or at least not in Standard English.**

**Response:** After the major revisions, we have the manuscript proofread and polished by a native English speaker specializing in medicine.
